# A Time-Frequency Domain Mixed Attention-Based Approach for Classifying Wood-Boring Insect Feeding Vibration Signals Using a Deep Learning Model

**DOI:** 10.3390/insects15040282

**Published:** 2024-04-16

**Authors:** Weizheng Jiang, Zhibo Chen, Haiyan Zhang

**Affiliations:** 1 School of Information Science and Technology, Beijing Forestry University, Beijing 100083, China; jiangweizheng@bjfu.edu.cn (W.J.); zhyzml@bjfu.edu.cn (H.Z.); 2 Engineering Research Center for Forestry-Oriented Intelligent Information Processing, National Forestry and Grassland Administration, Beijing 100083, China

**Keywords:** convolutional neural networks, holcocerus insularis staudinger, emerald ash borer, vibration signals, wood borer detection

## Abstract

**Simple Summary:**

To achieve targeted control of different wood-boring pests and targeted prevention of exotic invasions, it is necessary to classify the species of wood-boring pests. This paper collects feeding vibration signals of wood-boring pests using piezoelectric ceramic sensors and constructs a dataset. By applying attention mechanisms in both the time domain and the frequency domain, we design a novel CNN architecture named Residual Mixed Domain Attention Module Network (RMAMNet). RMAMNet significantly improves the accuracy of wood-boring pest classification, indicating its potential for effective pest monitoring and classification tasks. This study provides a new perspective and technical support for the automatic detection, classification, and early warning of wood-boring pests in forestry.

**Abstract:**

Wood borers, such as the emerald ash borer and holcocerus insularis staudinger, pose a significant threat to forest ecosystems, causing damage to trees and impacting biodiversity. This paper proposes a neural network for detecting and classifying wood borers based on their feeding vibration signals. We utilize piezoelectric ceramic sensors to collect drilling vibration signals and introduce a novel convolutional neural network (CNN) architecture named Residual Mixed Domain Attention Module Network (RMAMNet).The RMAMNet employs both channel-domain attention and time-domain attention mechanisms to enhance the network’s capability to learn meaningful features. The proposed system outperforms established networks, such as ResNet and VGG, achieving a recognition accuracy of 95.34% and an F1 score of 0.95. Our findings demonstrate that RMAMNet significantly improves the accuracy of wood borer classification, indicating its potential for effective pest monitoring and classification tasks. This study provides a new perspective and technical support for the automatic detection, classification, and early warning of wood-boring pests in forestry.

## 1. Introduction

Forests play a crucial role in sustaining life on Earth [1], ensuring human livelihoods and income. Forestry aims to maximize economic benefits while ensuring the continuous provision of ecological, social, and cultural services [2]. However, sustainability is threatened by various factors, including biological, non-biological, and anthropogenic elements [3]. The protection of forestry resources requires addressing the prevention and control of pests and diseases as a primary concern.

Among harmful insects, wood borers, with their concealed lifestyle, prolonged harmful period, and delayed manifestation of damage, pose the most challenging obstacles in pest control in forestry. Among all biological factors, wood borers and pathogens pose an increasingly severe threat to the integrity of forest ecosystems [4,5,6,7]. Two major wood borers, the emerald ash borer (EAB) and holcocerus insularis staudinger (*H. insularis*), parasitic on ash trees, are particularly challenging due to their concealed larval activity within tree trunks. Their damage is often inconspicuous until trees show evident symptoms, making intervention difficult [8,9,10].

In China, monoculture in artificial forests has led to a simplified forest structure, making them highly susceptible to extensive mortality in the event of an outbreak of wood-boring pests. Additionally, the continuous growth in international shipping has facilitated the introduction of numerous non-native species globally [11]. These pest larvae hide in wooden packaging, logs, and live plants, frequently moving between their native biogeographical regions and continents. Therefore, practical strategies need to be developed to detect potential invasive pests at an early stage [12].

EAB, originating from East Asia, was first discovered in southeastern Michigan and Windsor, Ontario in 2002, with such devastating consequences that it caused the death of local ash trees. Similar to several other invasive forest pests, EAB is likely introduced through international trade routes and established itself in highly urbanized environments with abundant hosts. In both urban and forested areas, the destructive impact of EAB is significant, resulting in the death of up to 15 million ash trees [9]. To prevent further spread, strict quarantine measures have been implemented in the United States and Canada, restricting the movement of ash trees, logs, and firewood. Meanwhile, although China has introduced American ash trees as ornamental plants, these introduced varieties lack resistance to EAB found in native Chinese ash trees, posing a potential threat.

Within China, EAB is primarily distributed in northern regions, with variations in its life cycle due to temperature differences. In some areas, there is one generation per year, while in others, it occurs biennially. Specifically, EAB has spread across Heilongjiang, Jilin, Liaoning, Shandong, Inner Mongolia, Hebei, Tianjin, and Beijing. However, it is noteworthy that in 2015, the first observation of the narrow-necked longhorn beetle occurred in Xinjiang, indicating an expansion of its distribution range [13].

Meanwhile, *H. insularis* also has a wide distribution in China, including Beijing, Tianjin, Hebei, Shandong, Jiangsu, Anhui, Jiangxi, Fujian, Hunan, Liaoning, Jilin, Heilongjiang, Inner Mongolia, Shaanxi, and Ningxia. Of concern is that, in 2023, *H. insularis* was recorded for the first time in Xinjiang, further demonstrating the trend of these wood-boring pests spreading and expanding within China [14].

**Figure 1:**
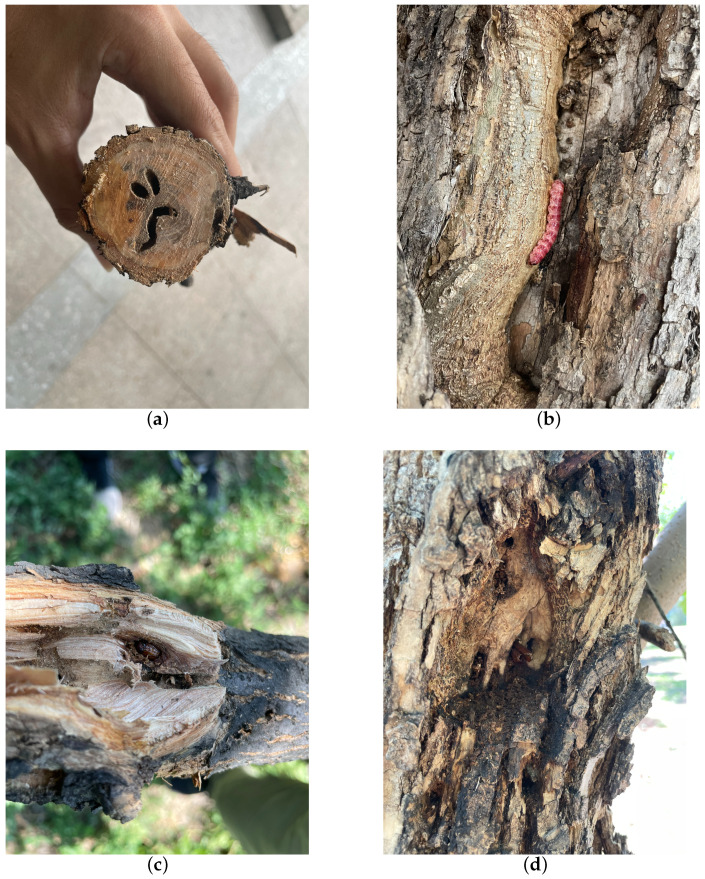
The traces left on ash trees as a result of the feeding activities of the *H. insularis*. (**a**) Wood borer cavities observed after the truncation of the tree trunk; (**b**) the emergence of *H. insularis* from the tree trunk; (**c**) the pupa within the tree trunk; (**d**) the pupal exuviae left behind after the eclosion of *H. insularis*.

*H. insularis* is a major wood borer affecting various tree species such as ash trees, Chinese parasol, purple-leaved plum, Chinese scholar tree, and willow. Its entire larval period involves feeding on the woody tissues inside tree trunks, resulting in significant harm to branches and even causing the breakage of tree trunks [15,16,17]; following the truncation of the tree trunk, wood borer cavities become visible within the central region of the trunk, as shown in Figure 1a. The adult *H. insularis* has a body length of approximately 24 mm, wingspan of about 50 mm, gray-brown color, and numerous black short lines on its wings. The ovum is elliptical, blocky, black-brown, with a network pattern on the surface. The eggs are laid in bark cracks or scars. Mature larvae can reach a length of 40 mm, with a bright red dorsal surface and a yellowish abdomen, as shown in Figure 1b. The pupa is of the obtect type, initially yellow-brown and gradually darkening, with spine rows on the abdomen and a prolonged pupal stage lasting about 20 days, as shown in Figure 1c. The larvae overwinter within tree tunnels, and in the North China region, mature larvae pupate in late May of the third year, with larval activity resuming in April of the following year. As temperatures rise, larval feeding increases dramatically, with May being the peak period of infestation. Larvae continue feeding within the tree trunk until October, producing significant amounts of wood shavings expelled from the trunk. The emergence period for adults occurs annually from June to August. During adult emergence, the pupal exoskeleton is partially exposed outside the emergence hole, as shown in Figure 1d. During this period, when tree trunks are cut, extensive damage is observed, with numerous red-colored larvae of different instars. The trunk is riddled with tunnels, and the larvae exhibit gregarious behavior, congregating near the cambium layer of the trunk for feeding. *H. insularis* has overlapping generations, a wide range of hosts, strong starvation resistance, and exhibits covert feeding behavior [18]. Due to the entirety of the *H. insularis* larval stage occurring within the tree trunk and pupation also taking place within the trunk, the pest is highly cryptic [19]. Controlling this pest is exceptionally challenging, and conventional control methods are often ineffective. For such pests, utilizing sex pheromones for pest management has become a convenient and effective approach.

**Figure 2:**
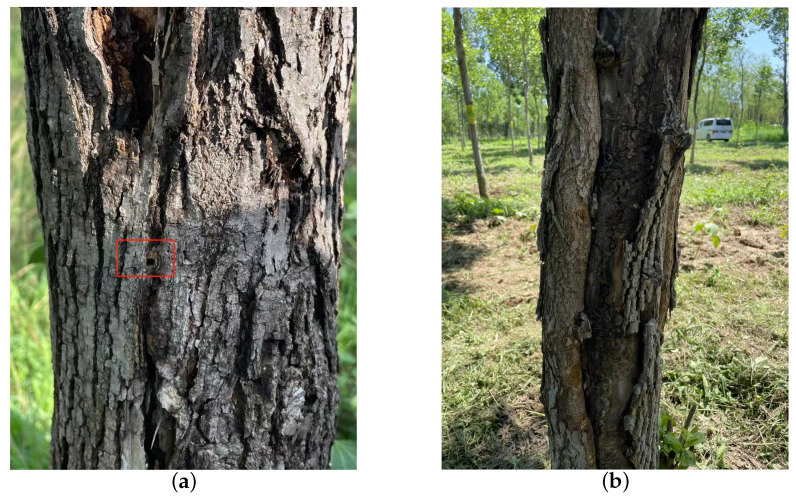
The traces left on ash trees as a result of the feeding activities of the EAB. (**a**) The D-shaped emergence holes left by the adult EAB following eclosion; (**b**) the longitudinal fissures resulting from prolonged feeding by the EAB and the galleries left by its wood-boring activity over the years.

EAB is the primary wood borer of ash trees, with all larval and pupal growth and development taking place inside tree trunks. The larvae feed within the cambium layer, creating wide, flat, and winding galleries that gradually weaken the tree. EAB larvae are flat, semi-transparent, milky white with a slight brownish tinge, and mature larvae are 34–45 mm in length. EAB infestation is highly concealed, and its natural mortality is low [14]. Typically, one generation occurs per year, with EAB activity starting in mid-April during the sap flow period in North China. Adults begin emerging in mid-May, reaching their peak emergence from late May to early June. After emerging, adults stay in the pupal chamber for 5–15 days before breaking through and forming a “D”-shaped emergence hole, as shown in Figure 2a. Newly emerged adults rely on leaf feeding for about a week to supplement nutrients. From mid-June to mid-July, adults mate and lay eggs on well-exposed dry bark and in crevices, with each female laying 68–90 eggs singly. The egg stage lasts 7–9 days. Larvae hatch in late June, burrow into the cambium and superficial layers of the woody tissue, disrupt the conductive tissues, and cause wilting or even death of the entire tree. In mid-October, EAB larvae start overwintering in galleries between the wood and phloem. The larval galleries are difficult to detect externally due to the absence of excreted frass until adults emerge, leaving behind characteristic “D”-shaped exit holes. As adult flight capabilities are limited, typically not exceeding 8–12 m, newly emerged adults tend to lay eggs on the same or nearby trees, exacerbating the damage over successive years. A continuous occurrence of EAB for 1–3 years in a forest can result in the death of perennial trees, as shown in Figure 2b.

Conventional approaches to pest detection, such as manual inspections within defined sample plots and the use of pheromone trapping techniques, including color-attractant traps, girdled-tree semiochemical attractant traps, and pheromone traps [20], along with remote sensing [21] and image detection, provide valuable information for pest control. However, these methods may not be sufficiently effective in early pest detection, especially for pests like the EAB and *H. insularis*, whose larvae cause significant damage to the host without conspicuous symptoms [22]. The delayed response characteristics of EAB damage, coupled with the challenges of early detection with the naked eye, can result in irreparable losses [23]. Consequently, the advancement of sound recognition technology has introduced new avenues for pest recognition.

For instance, researchers have utilized the AED-2000 to capture the activity sounds of the Red Palm Weevil and the AED-2010 equipment with an SP-1L probe to monitor the Grape Root Moth [24,25,26,27,28]. Presently, a convenient and rapid detection method involves using the AED instrument to collect tree vibrations, inputting these data into a deep learning model, and utilizing a neural network to identify insect infestations, particularly in the larval stage, thereby reducing the need for manual judgment [25]. Additionally, advancements in neural networks have given rise to pest monitoring and recognition based on speech recognition technology. Compared to early methods of recording feeding sounds in the air using fixed sensors on tree trunks, the piezoelectric vibration sounds generated by pest activity are more sensitive and less noisy. These vibrations can be recorded by sensors, saved in audio format, and identified and classified using deep learning technology [29,30]. In a study [31], parasitoids of the EAB, Tetrastichus planipennisi and Spathius agrili, determined the host’s weight and condition based on the frequency and amplitude of feeding vibrations, showcasing the potential to differentiate the sizes of wood-boring pests based on these differences. Thus, it is plausible to utilize the vibrational signals generated by feeding sounds for AI-based recognition of both the EAB and *H. insularis*, considering their distinctive morphologies.

Regarding *H. insularis* and EAB, research indicates that they can either individually or simultaneously infest a single ash tree [14]. Moreover, their control methods differ, with EAB typically managed using natural enemies such as parasitic wasps like Spathius agrili and Tetrastichus planipennisi, which are not effective against *H. insularis*. Due to the expensive nature of drugs targeting wood borer larvae and the distinct medications used for each type of wood borer, preventing the further spread of cryptic wood-boring pests through international trade and subsequent biological invasions underscores the critical importance of rapid and accurate classification and recognition. Consequently, in the absence of a dedicated classification and recognition model for wood-boring pests, we propose RMAMNet, an AI model based on the time-frequency domain, specifically designed for classifying wood borers in tree trunks.

## 2. Related Work

The recognition of pest vibrational signals involves two main stages. The first stage entails the auditory recognition and assessment of feeding vibrational signals produced by pests in the time-frequency domain, a task that demands specialized skills from detection personnel. Moreover, in environments with significant noise levels, short-duration recognition may lead to misjudgments. The second stage involves the collection of pest sounds or vibrational signals, followed by pest recognition through the use of the Internet of Things (IoT) and algorithms [32,33]. Machine learning stands out as a crucial automated recognition method, leveraging large datasets to discern patterns from raw data and subsequently classifying and predicting new input data. For instance, Sutin et al. [34] designed an algorithm for automatically detecting pulses generated by Anoplophora glabripennis and Agrilus planipennis larvae, using parameters associated with typical signals caused by larvae. When the detected pulse signal exceeds a specific threshold, it indicates the infection of trees.

Neural network algorithms, widely applied in machine learning, have witnessed rapid development in recent years, with artificial intelligence proving effective in addressing complex tasks and professional responsibilities [35]. Zhu et al. [36] utilized existing voice recognition parameterization techniques to identify pests. In their study, preprocessed audio data were used to extract Mel-scale Frequency Cepstral Coefficients (MFCCs), followed by classification using a trained Gaussian Mixture Model (GMM). Sun et al. [37] proposed a lightweight convolutional neural network, consisting of four convolutional layers and employing keyword discovery technology for the recognition of vibrations produced by Semanotus bifasciatus and Eucryptorrhynchus brandti larvae.

Currently, research in the field of pest vibrational signal classification and recognition is relatively limited. As automation technology continues to advance, the interest of researchers in pest vibrational signal classification and recognition techniques has grown. Beyond providing a basis for pest classification and recognition, pest vibrational signal recognition technology can play a significant role in pest control. However, the classification and recognition efficiency of various methods in the pest vibrational signal classification field has not been thoroughly explored. This study focuses on the activity vibrations of *H. insularis* and EAB. We utilize convolutional neural networks to extract features of pests and identify their presence in tree trunks. This approach aims to advance the understanding and application of pest vibrational signal recognition in the context of wood borers.

## 3. Materials and Methods

### 3.1. Dataset and Processing

#### 3.1.1. EAB Vibration Signal Collection

Due to the fact that the vibration signals used to identify *H. insularis* and EAB primarily arise from the subtle vibrations caused by the feeding activity of their larvae on the cambium layer of trees, the probing sensors need to be embedded into the trees during the signal collection process. Voltage-type sensors are employed to collect the vibration signals and save them in the form of single-channel audio. The sensor, jointly developed by Beijing Forestry University and Beihang University, allows the computer to receive signals from the sensor. These signals are then saved in waveform format using Audacity, with synchronous monitoring during the recording process. Embedding the collection probes into the tree trunks not only simplifies the information collection process but also, to some extent, reduces interference from environmental noise, enhancing the purity of the *H. insularis* and EAB larval vibrations.

To facilitate training and ensure the representativeness of the extracted feature, this study adopts two rounds of data collection, resulting in eight types of data that are organized into four classes for classification. The first collection occurred in 2021, targeting EAB larval feeding vibration signals, yielding four types of data; the first consists of EAB larval vibration signals collected from the field with ambient noise, the second comprises ambient noise collected using the same method as the EAB larval vibration signal collection, the third involves vibration signals from wood sections with EAB larvae in a soundproof indoor environment, and the fourth encompasses tree noise from wood sections without EAB larvae in a soundproof indoor environment. The ratios of ambient noise to signal for these two categories are set at 2:1. Signals collected outside the field for each type are obtained from the same tree at different heights and locations to ensure the diversity of vibration signal categories.

The second collection occurred in 2023, targeting *H. insularis* larval feeding vibration signals, using the same method as the EAB larval vibration signal collection. The data composition is identical to the first collection, yielding four types of data; the first consists of *H. insularis* larval vibration signals collected from the field in 2023 with ambient noise, the second comprises ambient noise collected using the same method as the *H. insularis* larval vibration signal collection, the third involves vibration signals from wood sections with *H. insularis* larvae in a soundproof indoor environment, and the fourth encompasses tree noise from wood sections without *H. insularis* larvae in a soundproof indoor environment. Similar to the first collection, the ratios of ambient noise to signal for these two categories are set at 2:1. Signals collected outside the field for each type ensure the diversity of vibration signal categories.

**Figure 3:**
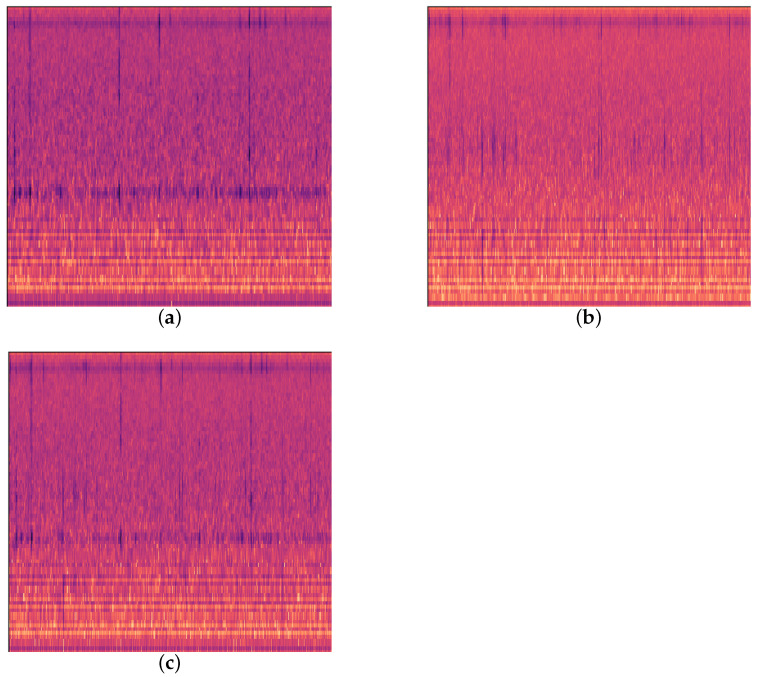
Three kinds of feeding vibration signals. (**a**) EAB feeding vibration signal; (**b**) *H. insularis* feeding vibration signal; (**c**) computer-synthesized signal simulation.

Since previous studies have found instances of both EAB and *H. insularis* appearing on the same ash tree [14], yet we did not collect feeding vibration signals indicating the simultaneous presence of both wood-boring pests on the same ash tree during our two data collections, in order to address this real-world scenario, we chose to synthesize new audio by overlaying the feeding vibration signals of the two pests in a 1:1 ratio using a computer as shown in Figure 3. We refer to these as “computer-synthesized signals simulating”.

The combined dataset comprises four classes: pure EAB larval feeding vibration signals from the field with ambient noise, pure *H. insularis* larval feeding vibration signals from the field with ambient noise, computer-synthesized signals simulating the presence of both EAB and *H. insularis* larvae feeding simultaneously, and ambient noise from both 2021 and 2023 collections. Given the potential scenario of EAB and *H. insularis* larvae parasitizing different parts of the same white poplar tree, the labeling of the data is performed manually after the completion of audio recording, based on visual inspection of the tree trunk. In Beijing, EAB larvae hatch in June, with their active stage extending from July to mid-August. *H. insularis* mature larvae undergo pupation at the end of July, with emergence occurring from late July to mid-August.

**Figure 4:**
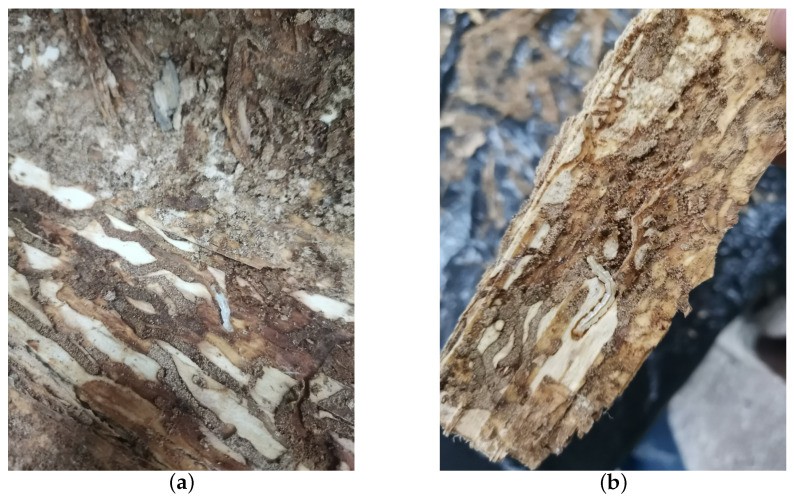
EAB larvae on a section of wood. (**a**) EAB larvae feeding on the phloem; (**b**) EAB larvae feeding on the phloem visible after removing the bark.

The vibrational data of EAB larval feeding were collected on 18 July 2021, with an average temperature of approximately 33 degrees Celsius over five consecutive days in a forest area in Tongzhou District, Beijing, affected by EAB larvae. Initially, tree observations were conducted to identify trees exhibiting significantly poorer growth compared to others. Trees were further inspected for the presence of “D”-shaped emergence holes, indicative of EAB infestation, without evidence of wood shavings resulting from *H. insularis* larval feeding. Piezoelectric vibration sensors’ probes were then inserted into the tree trunk sections. Prior to recording, manual monitoring of larval activity through headphones was performed. If larvae were still active in each trunk, recording sessions lasted for an hour and a half each day. Subsequently, the felled trees were transported to a soundproof indoor environment where the absence of larval feeding produced holes, created by *H. insularis* larvae. The temperature in the soundproof indoor environment was maintained at approximately 28 degrees Celsius. In total, six tree sections containing EAB larval vibration signals were collected. The setup included a computer, probe, and tree sections on a standard table. Before recording, larval activity was monitored through headphones to confirm their activity. Upon commencement of recording, all personnel left the room. For live tree trunks, if larvae remained active inside, vibration data were recorded for an hour and a half daily. Multiple audio segments were recorded for different parts of a trunk at various times. Two additional tree sections without EAB larvae, representing the background noise of the instruments, were included in the dataset. Each of these sections, with a length of approximately 30 cm, had the detector embedded at the midpoint of the tree section. Recording commenced on 23 July 2021, and spanned five days. After recording, under the supervision of forestry professionals, the bark was peeled off from all tree sections to reveal EAB larvae, which were then counted. Approximately 5 to 10 larvae were found in two tree sections, while the remaining four tree sections each contained around 10 to 20 larvae. The larvae measured approximately 3 cm in length, as shown in Figure 4a,b.

**Figure 5:**
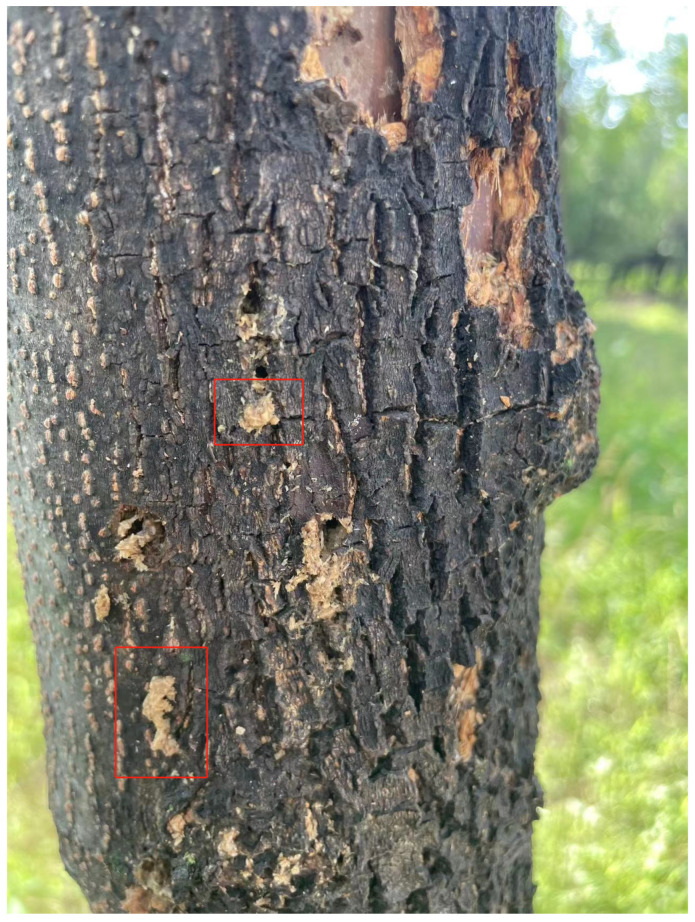
The wood shavings generated by the boring activity of *H. insularis*.

The vibrational data of *H. insularis* larvae during feeding were collected on 24 July 2023, with an average temperature of approximately 34 degrees Celsius. The data collection took place over five consecutive days in a forest area in Shunyi District, Beijing, known for infestations by both *H. insularis* and EAB larvae. Initially, tree observations were conducted to identify trees exhibiting notably poor growth compared to others. The presence of wood shavings resulting from *H. insularis* larval feeding was examined as a method to pinpoint ash trees highly likely to be infested by *H. insularis* larvae, as shown in Figure 5. Additionally, the absence of “D”-shaped emergence holes on the trees was verified. Subsequently, piezoelectric vibration sensors’ probes were inserted into the cross-sections of tree trunks for data collection.

Prior to recording, manual monitoring of larval activity was conducted using headphones. If larvae were found to be active within each tree trunk, recording sessions lasted for an hour and a half each day. Following this, the trees were felled, and the trunk sections were transported to a soundproofed indoor environment. Observation of the cross-sections of the felled trunks revealed characteristic boreholes resulting from the feeding activity of *H. insularis* larvae. The indoor environment maintained a temperature of approximately 28 degrees Celsius. A total of four tree sections, each containing vibrational signals from *H. insularis* larvae, were collected. The setup included a computer, probes, and tree trunks placed on an ordinary table.

**Figure 6:**
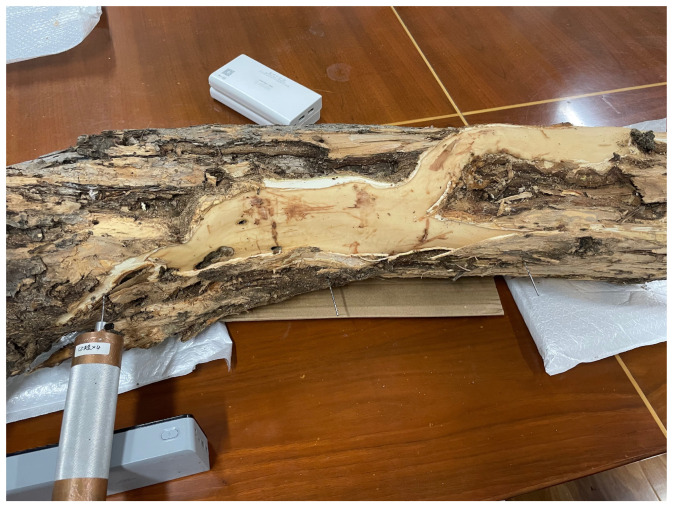
Collecting vibrational signals from *H. insularis* wood-feeding activity in a laboratory setting.

**Figure 7:**
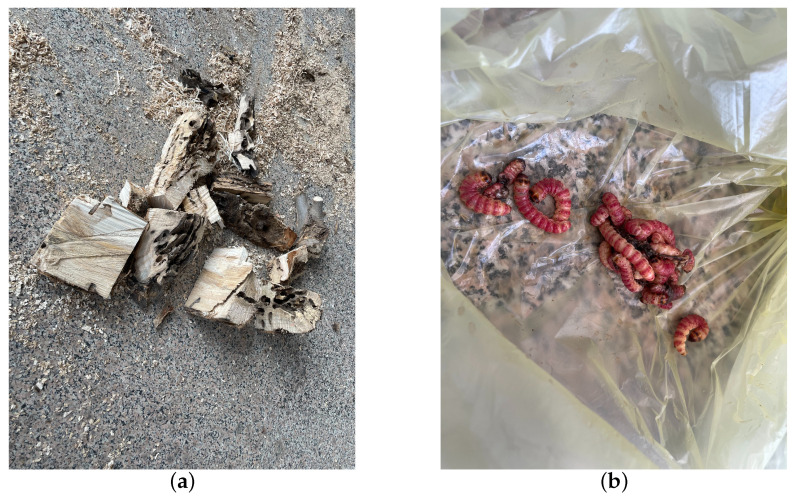
Quantification of *H. insularis* after the conclusion of vibration signal recordings. (**a**) Splitting wood sections for the purpose of quantifying the population of *H. insularis*; (**b**) portions of *H. insularis* larvae found within the wood section.

Preceding the recording sessions, headphones were employed to monitor larval activity for confirmation of their vitality. Once recording commenced, all personnel exited the room. For live tree trunks with active larvae, vibrational data were recorded daily for half-hour intervals. Specifically, multiple audio segments were recorded at different times for various sections of each tree trunk. As a control, the dataset included two tree sections without *H. insularis* larvae activity, primarily comprising background noise from the instruments. Each of these sections was approximately 30 cm in length, with the detector embedded in the midpoint of the tree trunk section. Recording began on 30 July 2023, and continued for five days. After recording concluded, under the supervision of forestry professionals, the bark was peeled off all sections of the tree trunk, revealing the absence of galleries produced by EAB larval feeding, as shown in Figure 6. The trunk was split open, and *H. insularis* larvae were counted, confirming the purity of the *H. insularis* larval feeding vibrational signals. In two tree sections, there were approximately 5 to 10 larvae and 1–3 pupae, while the other four tree sections each contained around 10 to 20 larvae and 3-5 pupae. Additionally, 28 *H. insularis* adult beetles were captured in the laboratory. The larvae measured approximately 4 cm in length, as depicted in Figure 7a,b.

Analysis of the collected vibration signals revealed characteristics such as short-term high energy, intervals, brief duration, and sharpness. The signals comprised multiple discrete pulses, with observed sawtooth waveforms and irregular intervals between pulses. The pulse waveforms exhibited a rapid increase to maximum amplitude, followed by a gradual smoothing, possibly due to irregular changes in the propagation of sound within the wood sections. These observations suggested that the activity sound was a complex wave formed by the superposition of multiple sine waves.

For both EAB larval and *H. insularis* larval feeding vibration signals with ambient noise from the collection site, audio segments with clear EAB and *H. insularis* larval feeding signals were selected through manual screening. In order to meet the independence conditions of the training and testing sets as much as possible, we conducted conditional random partitioning of the allocation of the training and testing sets. For two types of wood borers, we collected them in the wild for 5 days and indoors for 5 days, each time for an hour and a half. As the data collection process is accompanied by the growth, emergence, and death of larvae, theoretically, the vibration signals of wood borers will be different every day. In order to use more data for training as much as possible, we need to use the data every day as much as possible. Then, for the division of the training and testing sets, we divide the daily data into the first 30 min, middle 30 min, and last 30 min. Then, we take out the first 30 min and middle 30 min of each day as the range for selecting the training set, and the last 30 min of each day as the range for selecting the testing set. We cropped the audio within the designated ranges (the first 60 min of each piece of wood or tree per day as the selection range for the training set and the last 30 min as the selection range for the testing set) into audio segments of 5 s each. Then, we randomly selected audio segments from these cropped segments according to a ratio of 5:1 for the training set and testing set, respectively. For environment noise signals, the difference in acquisition time does not lead to differences in learned features, so we also divide them in the same way. Labels were assigned as follows: 0 for EAB larval feeding vibration signals, 1 for *H. insularis* larval feeding vibration signals, 2 for computer-synthesized EAB and *H. insularis* larval feeding vibration signals, and 3 for ambient noise collected during the two sessions. The data are presented in Table 1. Spectrograms for the first three types of signals are depicted in the figures. Detailed examination of the audio using Adobe Audition software revealed that the energy distribution frequency of EAB larval feeding data predominantly concentrated around the 17.5 kHz frequency range, while *H. insularis* larval feeding data predominantly focused around the 10.0 kHz frequency range.

We use a voltage sensor probe with a sampling frequency of 44.1 kHz and a sampling accuracy of 16 bits for signal acquisition. The probe is inserted into the tree trunk to capture the vibration signal directly.

#### 3.1.2. Preprocessing of Vibration Signal

Like many commonly used methods, we employed the Mel spectrogram to simulate the human ear’s perception of sound and extract features from audio segments. Since the vibrational signal of EAB larvae is a non-periodic signal that changes over time, fast Fourier transform, specifically short-time Fourier transform, is applied to obtain spectrograms over several windows of the new signal. Studies have shown that humans do not perceive frequency in a linear scale; instead, they are more adept at detecting differences in lower frequencies than higher frequencies. The mathematical operations on frequencies that result in the Mel scale align more closely with the human ear’s perception of frequency differences. Hence, for this study, which aims to mimic the human ear’s capture of EAB larval vibrational signals, the Mel spectrogram was chosen.

Firstly, before converting the vibrational signal to the Mel spectrogram, a sampling rate of 16 kHz is set, and the vibrational signal is randomly cropped for training data to audio segments of length less than or equal to 3 s. Any segments shorter than 3 s are zero-padded to reach the 3-s duration. Subsequently, the vibrational signal undergoes pre-emphasis. The input signal is then framed with a frame length of 512 and a step size of 160. The frames are separated using a Hanning window to ensure that no spectral information is lost. Short-time Fourier transform is applied to each frame signal, and the square of the absolute values is obtained. Finally, filtering is performed through 80 sets of Mel filters, followed by discrete cosine transform to obtain the final features.

**Figure 8:**
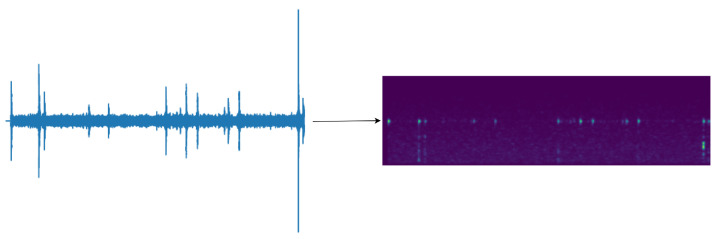
Transformation of vibration signals with random cropping to 301 × 80 Mel feature maps.

The feature size is 80 × 301, as shown in Figure 8, with the transformed time frames (301) serving as the feature length and the 80 sets of Mel filter groups as the number of channels. This conversion condenses the features from a two-dimensional representation to a one-dimensional feature, effectively leveraging the temporal and spectral information contained within the Mel filter groups. In presenting our work, for human comprehension convenience, we output the feature map as a spectrogram. This process does not compromise the original feature’s time and frequency domain information.

In order to achieve the classification of pest feeding vibration signals, our focus lies in the temporal and spectral distinctions of the audio. We propose a novel convolutional neural network (CNN) architecture based on the Residual Mixed Domain Attention Module (RMAM), which exhibits outstanding feature learning capabilities and scalability. To address the challenges posed by intra-class variability and inter-class similarity introduced by feeding vibration signals, our network incorporates channel-wise attention and temporal attention mechanisms. These attention mechanisms enhance the network’s ability to learn meaningful features. Subsequently, a Global Average Pooling (GAP) layer and a classification layer with softmax activation are employed to aggregate the acquired features and generate diagnostic results.

### 3.2. Method Overview

#### 3.2.1. Residual Learning

Firstly, consider a simple CNN block composed of convolutional layers. Let us assume that these layers learn a mapping function denoted as H(x). Throughout the training process, these layers directly fit H(x). Here, a basic CNN block can be defined by Equation (1):

(1) $$\begin{array}{c} y=F(x,W) \end{array}$$

where *x* and *y* represent the input and output of the block, F(·) is the mapping function, and *W* represents the parameters learned by these layers.

The fundamental assumption of residual learning is that, instead of directly learning the complex function H(x) by convolutional layers, it is easier to learn its residual function, simplifying the network training process. In the residual learning architecture, these convolutional layers learn the residual function H(x)−x rather than directly fitting H(x). The definition of residual learning is shown in Equation (2):

(2) $$\begin{array}{c} y=F(x,W)+x \end{array}$$

where the function F(·) represents the residual mapping learned by the convolutional layers. The residual learning mechanism is integrated into the network through skip connections. Although introducing residual learning allows for deeper networks and improved learning capabilities, it is worthwhile to explore more lightweight and efficient approaches to enhance the discriminative feature learning capability of the model.

#### 3.2.2. Channel Domain Attention Learning

In a 1D convolutional neural network (CNN), a convolutional layer processes a 1D temporal signal v(t) as input, applying convolutional kernels to capture meaningful features and generate feature maps. During training, the CNN optimizes and updates the kernel parameters to enhance its feature learning capabilities. From a signal analysis perspective, a convolutional kernel is represented as a temporal function c(t), acting on the input signal v(t). The feature learning process of a CNN involves the temporal convolution of c(t) with v(t). This temporal convolution is analogous to frequency domain multiplication, where the convolutional kernel serves as a filter determining which frequency domain information is retained or discarded. Each convolutional layer consists of multiple filters designed to capture distinct frequency domain features from the input signal.

In deep CNNs, each layer comprises numerous convolutional kernels, making it challenging to collect sufficient data for effective optimization of all kernel parameters. Some learned features may be relevant for diagnosis, while others may be irrelevant, potentially impacting the network’s decision making. Consequently, it is difficult to discern the importance of individual convolutional kernels for a diagnostic task, as the model treats all kernels as having equal importance weights.

**Figure 9:**
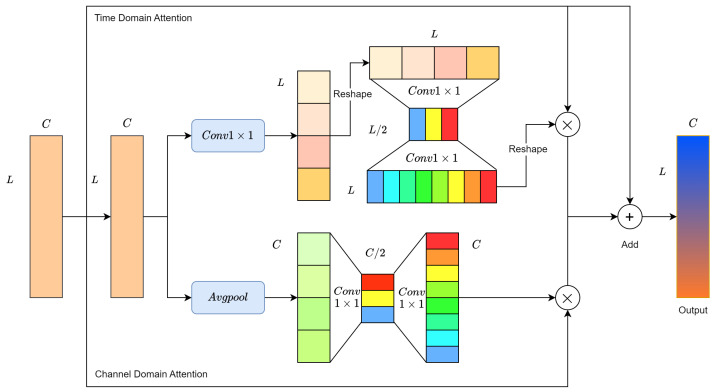
Residual Mixed Domain Attention Module.

To address this challenge, channel-wise attention is proposed. The Mel spectrogram is chosen as the input in a 1D form, where its channels represent frequency intervals. As illustrated in Figure 9, assume the output features of the second convolution module of RMAM (in the second row) are represented as $M=[m_{1},m_{2},\ldots ,m_{C}]$, where $m_{i}\in R^{L\times 1}$ denotes the feature of the *i*-th channel. First, a global average pooling layer aggregates global information in the temporal domain, producing a channel descriptor $z\in R^{1\times C}$ for each channel.

Channel-wise attention aims to identify which channel features are more crucial for pest recognition. Two non-linear transformation layers, each consisting of two 1 × 1 convolutional layers, are used to obtain the relative importance between different channels. The activation function α maps the resulting vector to a fixed weight range, generating the final channel weight vector $\hat{z}\in R^{1\times C}$. The values of $\hat{z}$ indicate the importance of the corresponding channel features. Finally, $\hat{z}$ is used to enhance the meaningful channel features in *M*, as shown in Equation (3).

(3) $$\begin{array}{c} N_{z}=M⊗\hat{z}=\left[m_{1}{\hat{z}}_{1},m_{2}{\hat{z}}_{2},\ldots ,m_{C}{\hat{z}}_{C}\right] \end{array}$$

Here, ${\hat{z}}_{i}$ is the *i*-th element of $\hat{z}$, and ⊗ denotes element-wise multiplication between two matrices.

#### 3.2.3. Time Domain Attention Learning

Vibration signals caused by wood-boring pests in infested wood are temporal signals containing both periodic and time-dependent information. Strong signal correlations exist between different time sequences, with valuable information concealed within certain signal sequences. For instance, wood-boring pests generate periodic pulses while gnawing on specific types of wood, inducing inherent frequencies. The components of the feeding vibration signal contain more meaningful information than other signal sequences, reflecting the intrinsic characteristics of wood-boring pests more directly. Therefore, the goal of the Time Domain Attention Learning Module is to make the network pay more attention to crucial signal sequences in the time domain.

As illustrated in Figure 9, we redefine *M* as $M=[m^{1},m^{2},\ldots ,m^{L}]$, where $m^{j}\in R^{1\times C}$ represents the feature at the *j*-th point on the time axis. We first use a 1×1 convolutional layer to aggregate global information in the channel domain, generating a time feature vector $q\in R^{L\times 1}$.

The essence of the time domain attention is to determine which information from signal segments is more crucial for wood-boring pest classification. For ease of convolutional calculations, the time feature vector $q\in R^{L\times 1}$ is adjusted to $q^{\prime }\in R^{1\times L}$. Similarly, two nonlinear layers are employed to encode the relative importance between time signal segments. Subsequently, the activation function α maps the obtained feature vector to a fixed weight range. Through reshaping operations, the final output is the time weight vector $\hat{q}\in R^{L\times 1}$. The value at a point in $\hat{q}$ indicates the importance of the corresponding time signal segment. Finally, $\hat{q}$ is used to enhance meaningful signal segment features in *M*, as shown in Equation (4).

(4) $$\begin{array}{c} N_{t}=M⊗\hat{q}=\left[m^{1}{\hat{q}}_{1},m^{2}{\hat{q}}_{2},\ldots ,m^{L}{\hat{q}}_{L}\right] \end{array}$$

where ${\hat{q}}_{j}$ represents the *j*-th element of $\hat{q}$.

#### 3.2.4. Mixed Domain Attention

The temporal domain attention module aims to extract signal components, such as pulses in the feeding vibration signal, relevant to the classification of wood-boring pests. On the other hand, the channel domain attention module focuses on extracting frequency components relevant to the classification of wood-boring pests. Therefore, the Residual Mixed Domain Attention Module (RMAM) simultaneously performs channel domain attention and temporal domain attention, combining the optimized features. This alleviates the impact of intra-class variability and inter-class similarity on the diagnostic performance of the network, thereby improving the performance of the wood-boring pest classification task.

The structure of RMAM is illustrated in Figure 9. After two convolutional modules, the output features are fed into two attention branches to enhance the network’s learning capability for meaningful features in both the channel and temporal domains. Lastly, the residual learning concept is introduced to ease the training difficulty of the network. In the majority of attention mechanism studies, the Sigmoid function is commonly used as the activation function α due to its generally good performance. We have chosen Sigmoid as the activation function for the network.

### 3.3. RMAMNet Architecture

**Figure 10:**
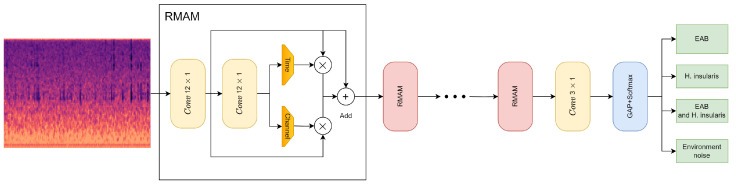
RMAMNet architecture.

As described above, this paper proposes the convolutional neural network RMAMNet, as shown in Figure 10. The network takes audio signals in the WAV format as input, featuring the main structure of RMAM convolutional layers, concatenated in four layers. After each RMAM layer, activation functions and pooling operations are applied. Finally, a linear classifier is connected to output the recognition results of the network.

The introduced RMA-CNN is a versatile and flexible end-to-end architecture for the classification of wood-boring insect vibration signals. By stacking RMAM, one can easily construct RMA-CNN architectures of arbitrary depth. In this experiment, we utilized a lightweight version, namely RMA-CNN-10 (indicating only ten learnable layers in the network). For simplicity, we use RMA-CNN to refer to RMA-CNN-10 in the following description. The structure and parameters of RMA-CNN are presented in Table 2.

To ensure that input signal samples contain complete signal cycles, the input dimension of RMA-CNN-10 is set to 2048 × 1. RMA-CNN consists of one convolutional module and one classification layer, which includes four RMAMs. Each convolutional module is composed of a 1D convolutional layer, a batch normalization layer, and a Sigmoid activation function. In order to capture features on longer signal segments, we used wider convolutional kernels in the first and second RMAM, with sizes of 12 × 1 and 6 × 1, respectively. The number of channels gradually increases from 80 to 256. We employed max-pooling techniques to reduce the dimensionality of features while retaining valuable information. In the classification stage, a GAP layer is used, and a fully connected layer with the Softmax function is employed to provide the final diagnostic results. Additionally, we constructed several pure CNNs as comparative methods for our approach. The pure CNNs also consist of ten learnable layers, including nine convolutional layers and one fully connected layer. There are methods with more than ten learnable layers to achieve higher recognition accuracy.

## 4. Recognition Process

**Figure 11:**
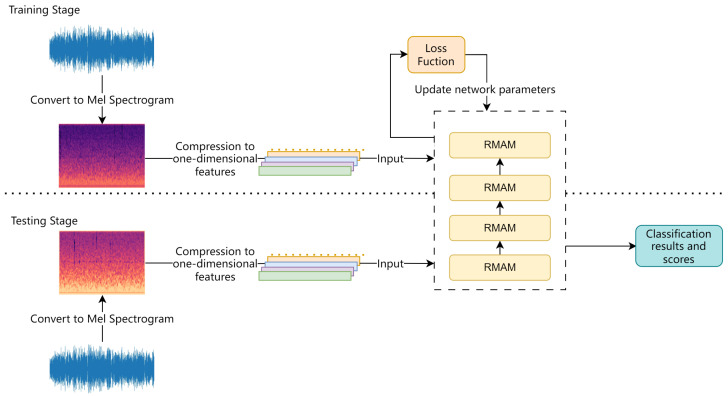
Flowchart of training and testing process.

The process of training and recognition in RMAMNet is illustrated in Figure 11. At the beginning of the network training stage, similar to conventional sound recognition networks, the logarithmic Mel spectrogram extracted during the preprocessing stage is used as the input for training. In this study, 80 Mel frequency spectrogram features are extracted from the audio data and uniformly processed into 301 × 80 image data, serving as the input data for the network. Initially, predictions for the categories are made through forward propagation. Subsequently, the loss function is employed to compute the error between the predicted categories and the true categories. Finally, the network parameters are updated through backpropagation.

To validate the accuracy of the model, during the testing phase, entirely different audio data from the training set are used. The network predicts categories based on the results of the neural network.

## 5. Experimental Results and Analysis

### 5.1. Experimental Environment

The study utilized an environment with an Intel(R) Xeon(R) Platinum 8255C 12 vCPU (43 GB memory) and a GeForce GTX 3080 (10 GB VRAM). The implementation was based on the PyTorch deep learning framework.

The process of setting hyperparameters involved both manual tuning and automatic tuning. Considering relevant experiments in audio recognition and the workload of this study, we manually adjusted hyperparameters. After conducting numerous experiments, we identified the optimal parameter values for performance and utilized them in the study. The batch size was set to 512, and the model training concluded after 12 epochs.

### 5.2. Experimental Results

The pest recognition is conducted on a per-audio basis, and the study employs audio recognition accuracy and F1 score as the final evaluation metrics for the model. When comparing the recognition performance of different networks, it is essential to preprocess the test audio, input it into the network model, and transform it into the logarithmic Mel sound feature spectrum. This process is foundational in audio recognition and is independent of the choice of recognition method.

To validate the recognition accuracy of RMAMNet, the study conducts comparative experiments with some established network structures. In convolutional neural networks, the ability to adapt to more complex functions increases with the number of layers. Although neural networks use backpropagation for weight updates, increasing the number of layers eventually leads to the vanishing gradient problem. ResNet’s shortcut connections are beneficial in addressing the issues of vanishing and exploding gradients, significantly improving recognition accuracy. In this study, to examine the effectiveness of the proposed network, ResNet, VGG, and other networks are introduced through transfer learning. However, for vibration signal recognition, a relatively simple network structure can achieve high accuracy [38]. The same dataset and data processing methods are used. The last layer of the network model is modified, setting it as a new classification layer. This ensures that the output dimension matches the number of classes in the dataset, and the network is then evaluated. Therefore, VGG and ResNet are employed as comparative networks to identify the feeding vibration signals of EAB larvae and *H. insularis* larvae.

The experimental results in Table 3 indicate that the recognition accuracy of RMAMNet is 95.34%, while ResNet10, ResNet18, VGG10, and VGG19 achieve recognition accuracies of 81.85%, 83.35%, 70.36%, and 75.46%, respectively. RMAMNet exhibits an F1 score of 0.95, outperforming ResNet10 (0.82), ResNet18 (0.83), VGG10 (0.71), and VGG19 (0.76).

**Figure 12:**
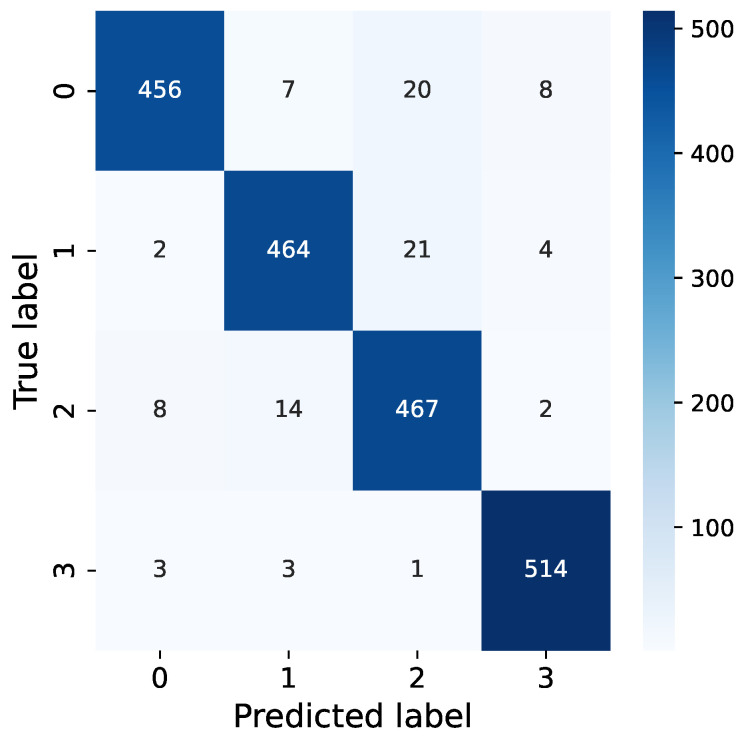
Confusion of our RMAMNet.

From our confusion matrix in Figure 12, it is evident that the most common misclassifications occur when dealing with synthetic vibration signals that combine both EAB and *H. insularis* feeding vibration signals. This is likely due to the weakening of vibration signals resulting from the pests’ distance from the sensor, as the distance between the sensor and the sound source significantly impacts the intensity of vibration signals. We hypothesize that when synthesizing signals, a strong feeding vibration signal from one pest type combined with a fainter signal from another pest type could lead to a reduced proportion of the fainter signal in combined vibration signals, potentially resulting in undetected instances. The neural network may mistakenly interpret this combination as a prominent feeding vibration signal mixed with environmental noise, causing misclassification. Additionally, synthetic vibration signals are more prone to being misclassified as pure *H. insularis* vibration signals, possibly because *H. insularis*, being larger in size than EAB, produces more prominent vibration signals, leading to misclassification during the classification process where the presence of *H. insularis* is detected but not EAB.

Furthermore, vibration signals from environmental noise are distinguishable from both *H. insularis* and EAB feeding vibration signals, but they are more likely to be misclassified as pure EAB feeding vibration signals. This could be attributed to some of the tested EAB activities occurring at a greater distance from the sensor, resulting in a higher level of ambient noise in the vicinity, thus leading to undetected EAB feeding vibration signals.

**Figure 13:**
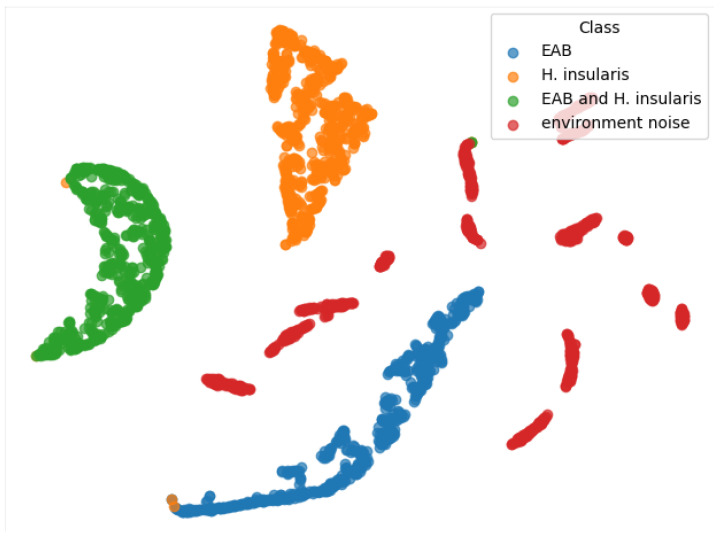
TSNE of our RMAMNet.

From the TSNE graph in Figure 13, we can see that all three types of feeding vibration signals are well-distinguished, and environmental noise is also beneficial for the three types of feeding vibration signals, but they are not clustered together. We conclude that the reason is that the environmental noise used for training has no obvious characteristics. Generally speaking, any vibration signal that does not belong to the three types of feeding vibration signals should belong to environmental noise. In an ideal state, environmental noise is a negative inference—a signal that does not contain vibration signals from boring pests.

The suboptimal performance observed when directly applying mature recognition networks in this experiment is evident from the experimental results. RMAMNet significantly outperforms other network models in terms of recognition accuracy and F1 score, indicating that the proposed RMAMNet achieves superior performance in the classification task of wood borer feeding vibration signals. In recognition and classification experiments, the performance of the network is often associated with the characteristics of the original dataset. Therefore, it is essential to flexibly adjust the recognition model based on the characteristics of the target dataset collected.

## 6. Discussion

Currently, in the recognition research of wood borers, the acquisition of raw data has been a major challenge due to the unique living environment of these pests. The lack of standardized collection criteria has resulted in very few publicly available datasets, significantly impeding the progress and development of wood borer recognition and classification technologies. In this study, we utilized self-developed piezoelectric ceramic sensors to collect feeding vibration signals from two types of pests and simulated three scenarios of feeding vibration signals. The use of sensors enables the monitoring of wood borer activity within trees, preserving signals without causing damage to the trees. This simple collection method provides valuable insights for other researchers and establishes a foundation for subsequent studies.

For the scenario where both pests coexist, our simulations may not necessarily reflect the real-world situation accurately. It is essential to validate the authenticity of our simulations through subsequent data collection efforts.

In the traditional field of speech recognition, some networks have demonstrated outstanding performance. From data-driven clustering to the optimization of residual structures, they have addressed degradation issues in neural networks. However, in this study, audio signal features are relatively limited, and the feature information is discontinuous, exhibiting intra-class variability and inter-class similarity. Existing models cannot effectively perform the monitoring task. Analyzing the experimental results from the above study, it can be concluded that, unlike voices or other sounds with distinctive features, vibration signals from different types of wood borers have strong characteristics. Irregular and intermittent audio frequencies are challenging to identify, but our temporal and frequency domain attention mechanisms effectively overcome these issues. The recognition network proposed in this study provides new research perspectives and technical support for the detection and classification of wood borers in forestry.

## 7. Conclusions

In this study, piezoelectric ceramic sensors were employed to collect feeding vibration signals from EAB larvae and *H. insularis* larvae. Simultaneously, outdoor and indoor ambient noise audio, along with synthesized signals, were collected to form a completely new dataset. Adjustments were made to the proposed network model based on the characteristics of pest sounds. Compared to recognition results from other networks, RMAMNet efficiently and accurately classifies pests. Therefore, the proposed method in this study is capable of adapting to the monitoring and classification tasks of wood-boring pests, providing technical support for the automatic monitoring, classification, and early warning recognition of wood-boring pests.

One limitation of our research lies in the insufficient data. The feeding vibration signals produced by the coexistence of two wood borers in real-life situations may differ from those generated when both species are artificially synthesized. In this study, we did not collect feeding vibration signals when both species coexist. Additionally, we lack data on whether wood borers of different ages and sizes within the same species produce feeding vibration signals different enough to affect the classification by our model.

Furthermore, regarding bioinvasion, the condition of wood which serves as export goods may change due to storage or other factors, potentially altering the feeding vibration signals produced by the insects within. Thus, whether the model trained on feeding vibration signals collected in our experimental environment can be directly applied to detecting wood borers in exported wood requires further experimentation.

In the future, we plan to collect vibration signals from different tree species, various pests, and different forest regions to further validate the feasibility of convolutional neural networks. We will pay more attention to information such as pest species and larval stages to assess the specific damage conditions of trees.

## Figures and Tables

**Table 1:** Dataset composition.

Categories	Representation	Duration (Min)	Sample Rate (kHz)	Sample Depth (bit)
EAB vibration signals	0	250	44.1	16
*H. insularis* vibration signals	1	250	44.1	16
computer-synthesized EAB and *H. insularis*	2	250	44.1	16
environmental noise	3	298	44.1	16

**Table 2:** Parameters and structure of RMA-CNN.

Layer	Type	Kernel/Channel	Stride/Padding	Output
1	RMAM	12 × 1/128	1/yes	123 × 128
2	Pooling	–	4/-	123 × 128
3	RMAM	6 × 1/256	1/yes	30 × 256
4	Pooling	–	4/-	30 × 256
5	RMAM	3 × 1/256	1/yes	16 × 256
6	Pooling	–	2/-	16 × 256
7	RMAM	3 × 1/256	1/yes	9 × 256
8	Pooling	–	2/-	9 × 256
9	Convolution	3 × 1/256	1/yes	4 × 256
10	Pooling	–	2/-	4 × 256
11	Global Average Pooling	-	-	256
12	Softmax	-	-	4

**Table 3:** Experimental results.

Model	Recognition Accuracy	F1 Score
RMAMNet	95.34%	0.95
ResNet10	81.85%	0.82
ResNet18	83.35%	0.83
VGG10	70.36%	0.71
VGG19	75.46%	0.76

## Data Availability

The raw data presented in this study are available on request from the corresponding author.
